# Genetic Characterization of Spondweni and Zika Viruses and Susceptibility of Geographically Distinct Strains of *Aedes aegypti*, *Aedes albopictus* and *Culex quinquefasciatus* (Diptera: Culicidae) to Spondweni Virus

**DOI:** 10.1371/journal.pntd.0005083

**Published:** 2016-10-26

**Authors:** Andrew D. Haddow, Farooq Nasar, Hilda Guzman, Alongkot Ponlawat, Richard G. Jarman, Robert B. Tesh, Scott C. Weaver

**Affiliations:** 1 The University of Texas Medical Branch, Institute for Human Infections and Immunity, Department of Pathology and Center for Biodefense and Emerging Infectious Diseases, Galveston, Texas; 2 United States Army Medical Research Institute of Infectious Diseases (USAMRIID), Virology Division, Fort Detrick, Maryland; 3 Armed Forces Research Institute of Medical Sciences (AFRIMS), Department of Entomology, Bangkok, Thailand; 4 Armed Forces Research Institute of Medical Sciences (AFRIMS), Department of Virology, Bangkok, Thailand; 5 The University of Texas Medical Branch, Institute for Human Infections and Immunity, Department of Microbiology & Immunology, Galveston, Texas; Duke-NUS GMS, SINGAPORE

## Abstract

**Background:**

Zika virus (ZIKV) has extended its known geographic distribution to the New World and is now responsible for severe clinical complications in a subset of patients. While substantial genetic and vector susceptibility data exist for ZIKV, less is known for the closest related flavivirus, Spondweni virus (SPONV). Both ZIKV and SPONV have been known to circulate in Africa since the mid-1900s, but neither has been genetically characterized by gene and compared in parallel. Furthermore, the susceptibility of peridomestic mosquito species incriminated or suspected in the transmission of ZIKV to SPONV was unknown.

**Methodology/Principal Findings:**

In this study, two geographically distinct strains of SPONV were genetically characterized and compared to nine genetically and geographically distinct ZIKV strains. Additionally, the susceptibility of both SPONV strains was determined in three mosquito species. The open reading frame (ORF) of the SPONV 1952 Nigerian Chuku strain, exhibited a nucleotide and amino acid identity of 97.8% and 99.2%, respectively, when compared to the SPONV 1954 prototype South African SA Ar 94 strain. The ORF of the SPONV Chuku strain exhibited a nucleotide and amino acid identity that ranged from 68.3% to 69.0% and 74.6% to 75.0%, respectively, when compared to nine geographically and genetically distinct strains of ZIKV. The ORF of the nine African and Asian lineage ZIKV strains exhibited limited nucleotide divergence. *Aedes aegypti*, *Ae*. *albopictus* and *Culex quinquefasciatus* susceptibility and dissemination was low or non-existent following artificial infectious blood feeding of moderate doses of both SPONV strains.

**Conclusions/Significance:**

SPONV and ZIKV nucleotide and amino acid divergence coupled with differences in geographic distribution, ecology and vector species support previous reports that these viruses are separate species. Furthermore, the low degree of SPONV infection or dissemination in *Ae*. *albopictus*, *Ae*. *aegypti* and *Cx*. *quinquefasciatus* following exposure to two geographically and genetically distinct virus strains suggest a low potential for these species to serve as vectors.

## Author Summary

Spondweni virus (SPONV) is a mosquito-transmitted flavivirus reported in Africa. Human infection with SPONV may result in a febrile illness similar to symptomatic Zika virus (ZIKV) infection, as well as many other tropical infections. Previously, little was known about the genetic relationships between SPONV and ZIKV. Additionally, the ability of SPONV to infect peridomestic mosquito species suspected or incriminated in the transmission of ZIKV was unknown. Both SPONV strains exhibited a high degree of nucleotide and amino acid identity to each other, but considerable nucleotide and amino acid divergence to ZIKV. The open reading frame (ORF) of the nine African and Asian lineage ZIKV strains originally isolated in West Africa, Central Africa, East Africa, Southeast Asia, the Pacific Islands and the New World all exhibited limited nucleotide divergence. Both strains of SPONV exhibited a low degree of infection and/or dissemination in *Aedes albopictus*, *Ae*. *aegypti* and *Culex quinquefasciatus* mosquitoes suggesting that these species have a low potential to serve as vectors. These results coupled with differences in geographic distribution, ecology and vector species indicate that SPONV and ZIKV are similar but separate species.

## Introduction

The Spondweni serogroup, genus *Flavivirus* (*Flaviviridae*), includes two species–Zika virus (ZIKV) and Spondweni virus (SPONV) [1]. Both ZIKV and SPONV are associated with human illness [2,3]. SPONV can cause a self-limiting febrile illness characterized by headache, myalgia, nausea and arthralgia [3–7]; signs and symptoms similar to most reported symptomatic ZIKV infections [8–16], making diagnosis challenging in those regions of Africa with virus co-circulation. Although SPONV is not typically associated with serious disease, a subset of patients report signs and symptoms suggestive of vascular leakage and/or neurological involvement [3].

In 1952, the Chuku strain of SPONV was isolated from the blood of a febrile patient in Nigeria [6]. This strain was initially misclassified as ZIKV [3,17], leading to the 1955 South African SA Ar 94 *Mansonia uniformis* mosquito isolate being classified as the prototype SPONV strain [18]. Since its initial isolation, SPONV activity has been reported throughout sub-Saharan Africa (Table 1). In nature, the maintenance cycle is unclear [2,17,19], but may be similar to the non-human primate/mosquito cycle that is utilized by ZIKV [11,13]. Although SPONV has been isolated from several mosquito genera (Table 1), the vast majority of isolations have been made in the sylvatic mosquito, *Aedes circumluteolus* [7,20,21].

**Table 1:** Reported geographic distribution of Spondweni virus*.

Country	Seroprevelance^†^ (Humans)	Virus isolation (Human)	Virus isolation (Mosquito)	Reference(s)
Angola	X			[68]
Botswana	X			[69]
Burkina Faso	X			[70]
Cameroon	X	X	*Eretmapodites spp*.	[70,71]
Ethiopia	X			[72]
Gabon	X			[70]
Mozambique	X		*Aedes fryeri/fowleri*	[73]
Namibia	X			[69]
Nigeria	X	X		[6]
South Africa	X		*Ae*. *circumluteolus*, *Ae*. *cumminsi*, *Culex neavi*, *Cx*. *univittatus*, *Er*. *silvestris*, *Mansonia africana*, *Ma*. *uniformis*	[7,18,20,21]

*Does not include laboratory-acquired infections.

^†^ Seroprevalence was determined by one or more of the following methods: Hemagglutination inhibition, neutralization and/or complement-fixation.

It is possible due to antigenic cross-reactivity among flaviviruses that seropositive individuals may have been previously exposed to one or more flaviviruses and not to Spondweni virus.

Like other flaviviruses SPONV has a positive-sense single stranded RNA genome of approximately 11 kilobases in length [22]. The genome contains 5′ and 3′ untranslated regions flanking a single open reading frame (ORF) that encodes a polyprotein that is cleaved into three structural proteins: the capsid (C), premembrane/membrane (prM), and envelope (E), and seven non-structural proteins (NS1, NS2A, NS2B, NS3, NS4A, 2K, NS4B, and NS5) [22].

Herein, we genetically characterize two SPONV strains and investigate their potential for urban emergence as seen with ZIKV, as well as with other flaviviruses, including yellow fever and dengue viruses [23,24]. We determined the genetic relationship between the Nigerian Chuku and South African SA Ar 94 strains of SPONV and compared their sequence data to nine geographically and genetically distinct strains of ZIKV. We also determined the susceptibility of *Ae*. *aegypti*, *Ae*. *albopictus* and *Culex quinquefasciatus* mosquitoes to both strains of SPONV–mosquito species incriminated or suspected in transmitting ZIKV [25–28].

## Methods

### Virus strains and virus propagation

Virus strains were obtained from the World Reference Center of Emerging Viruses and Arboviruses Collection at the University of Texas Medical Branch in Galveston, Texas. Both the Nigerian Chuku and South African SA Ar 94 strains prior passage histories were unknown and therefore these viruses could exhibit passage-associated mutations. Both viruses were passaged once in *Ae*. *albopictus* cells (C6/36; ATCC #CCL-1660) for sequencing, and subsequently passaged once in African green monkey kidney cells (Vero; ATCC #CCL-81) for vector susceptibility experiments. Vero cells were maintained at 37°C in a total volume of 20 ml of media containing Dulbecco's Modified Eagle Medium (DMEM) (Gibco, Carlsbad, CA, USA) supplemented with 2% (vol/vol) fetal bovine serum (FBS), 100 U/ml of penicillin, 100 μg/mL of streptomycin, and 0.5 mg/ml amphotericin B (Sigma-Aldrich, St. Louis, MO, USA). C6/36 cells were maintained at 29°C in culture media that was also supplemented with 0.1 mM non-essential amino acids, 1.0 mM sodium pyruvate, and 1% tryptose phosphate broth (vol/vol) (Sigma-Aldrich, St. Louis, MO, USA). Following virus harvest, virus stocks were aliquoted and frozen at -80°C.

### RNA preparation, genomic amplification, and sequencing

Viral RNA was extracted from cell culture supernatant using the QIAamp Viral RNA Kit (Qiagen, Valencia, CA, USA). Overlapping primer pairs (S1 Table) were used to amplify the entire open reading frame (ORF) using the Titan OneStep RT-PCR kit (Roche, Mannheim, Germany) and purified amplicons were directly sequenced using the Applied Biosystems BigDye Terminator version 3.1 Cycle Sequencing Kit (Foster City, CA, USA) and the Applied Biosystems 3100 Genetic Analyzer (Foster City, CA, USA). Nucleotide sequences derived from both SPONV strains were assembled in Vector NTI Suite (Invitrogen, Carlsbad, CA, USA), aligned in SeaView [29] using MUSCLE [30], and edited in MacVector (Apex, NC, USA). These consensus sequences were deposited in GenBank, SPONV Chuku strain accession no. KX227369 and SPONV SA Ar 94 strain accession no. KX227370.

### Genetic analyses

ZIKV strains currently fall into either the African or Asian lineages [11,15]; as such nine geographically and genetically distinct sequences (i.e. strains) were used as representative members of these lineages for nucleotide and amino acid comparisons with both SPONV strains. The selected strains were isolated in West Africa (n = 1), Central Africa (n = 1), East Africa (n = 1), Southeast Asia (n = 2), the Pacific Islands (n = 2), and the New World (n = 2). These strains include the prototype strain MR 766 (Uganda 1947) GenBank accession no. AY632535 [22]; ArB 13565 (Central African Republic 1976) GenBank accession no. KF268948.1 [31]; ArD 41519 (Senegal 1984) GenBank accession no. HQ234501.1 [11]; P6-740 (Malaysia 1968) GenBank accession no. HQ234499 [11]; CPC-0740 (Philippines 2010) GenBank accession no. KM851038.1; EC Yap (Yap Island 2007) GenBank accession no. EU545988.1 [32]; H/FP/2013 (French Polynesia 2013) GenBank accession no. KJ776791.1 [33]; Z1106033 (Suriname 2015) GenBank accession no. KU312312.1 [34]; and PRVABC59 (Puerto Rico 2015) GenBank accession no. KU501215.1 [35]. The MR 766 sequence used in these analyses exhibited a deletion in the potential glycosylation site that has been previously noted [11,22].

### Mosquito rearing, maintenance, and artificial infectious blood feeds

Three laboratory colonized, geographically distinct strains of both *Ae*. *albopictus* and *Ae*. *aegypti*, and one strain of *Cx*. *quinquefasciatus* were used to determine mosquito susceptibility to both SPONV strains (Table 2). Mosquitoes were reared and maintained during experiments using a 12:12 hour light/dark photoperiod in approximately 80% relative humidity at 28°C, and adult mosquitoes were provided a 10% sucrose solution via a cotton ball. Four- to seven-day-old female mosquitoes were sugar starved for 24 hours prior to infectious blood meal feeding, with *Ae*. *albopictus* and *Cx*. *quinquefasciatus* having access to deionized water up to 12 hours prior to feeding to reduce physiological stress.

**Table 2:** Mosquito susceptibility to Spondweni virus Chuku and SA Ar 94 strains.

A. Susceptibility of selected mosquito species to Spondweni Chuku strain, dose 5.1 log_10_ PFU/mL.						
**Mosquito (origin)**	**Proportion infected**	**Percent infected**	**95% CI**	**Proportion with disseminated infection**	**Percent with disseminated infection**	**95% CI**
*Aedes aegypti* (Galveston, USA)	0/19	0.0	0.0, 16.8	0/19	0.0	0.0, 16.8
*Aedes aegypti* (Iquitos, Peru)	0/20	0.0	0.0, 16.1	0/20	0.0	0.0, 16.1
*Aedes aegypti* (Thailand)	0/4	0.0	0.0, 49.0	0/4	0.0	0.0, 49.0
*Aedes albopictus* (Galveston, USA)	0/24	0.0	0.0, 13.8	0/24	0.0	0.0, 13.8
*Aedes albopictus* (Thailand)	0/12	0.0	0.0, 24.3	0/12	0.0	0.0, 24.3
*Aedes albopictus* (Venezuela)	0/3	0.0	0.0, 56.2	0/3	0.0	0.0, 56.2
*Culex quinquefasciatus* (Galveston, USA)	0/24	0.0	0.0, 13.8	0/24	0.0	0.0, 13.8

B. Susceptibility of selected mosquito species to Spondweni SA Ar 94 strain, dose 5.3 log_10_ PFU/mL.						
**Mosquito (origin)**	**Proportion infected**	**Percent infected**	**95% CI**	**Proportion with disseminated infection**	**Percent with disseminated infection**	**95% CI**
*Aedes aegypti* (Galveston, USA)	0/24	0.0	0.0, 13.8	0/24	0.0	0.0, 13.8
*Aedes aegypti* (Iquitos, Peru)	0/24	0.0	0.0, 13.8	0/24	0.0	0.0, 13.8
*Aedes aegypti* (Thailand)	1/24	0.0	0.2, 20.2	0/24	0.0	0.0, 13.8
*Aedes albopictus* (Galveston, USA)	2/24	8.3	1.5, 25.9	2/24	8.3	1.5, 25.9
*Aedes albopictus* (Thailand)	3/24	12.5	4.3, 31.0	0/24	0.0	0.0, 13.8
*Aedes albopictus* (Venezuela)	0/24	0.0	0.0, 13.8	0/24	0.0	0.0, 13.8
*Culex quinquefasciatus* (Galveston, USA)	0/24	0.0	0.0, 13.8	0/24	0.0	0.0, 13.8

Mosquito infections were performed in an Arthropod Containment Level-3 (ACL3) laboratory following the guidelines set forth under the Biosafety in Microbiological and Biomedical Laboratories (BMBL) 5th Edition Appendix E (Arthropod Containment Guidelines). Groups of 100 mosquitoes were allowed to feed from artificial membrane feeders (Discovery Workshops, Lancashire, UK) covered by rat skins and containing a suspension of one part defibrinated sheep blood (Colorado Serum Company, Denver, CO, USA) and one part thawed virus. Virus titration was performed by plaque assay on infectious blood meals at 37°C using 90% confluent Vero cells in six-well plates with media containing Modified Eagle Medium (MEM) (Gibco, Carlsbad, CA, USA) supplemented with 10% FBS (vol/vol), 100 μg/mL of penicillin, 100 μg/ml of streptomycin, 0.1 mM non-essential amino acids, and 2 mM glutamine (Sigma-Aldrich, St. Louis, MO, USA). Serial 10-fold dilutions of infectious blood meals were inoculated onto the cell monolayer rinsed with phosphate-buffered saline (Gibco, Carlsbad, CA, USA). Virus was allowed to absorb for 30 min at room temperature after which the monolayer was overlaid with 4 mL of a 1:1 solution of 2% agar–2× MEM. 96 hours after the first overlay, 2 mL of a 1:1 solution of 2% agar–2× MEM containing 2% neutral red (Sigma-Aldrich, St. Louis, MO, USA) was added to each well. Plaques were counted at 120 hours, and infectivity titers were expressed as PFU/mL. Blood meal titers were 5.1 (Chuku strain) and 5.3 (SA Ar 94 strain) log_10_ PFU/mL. Post feeding, mosquitoes were sorted on ice and individuals meeting the criteria for stages 4 to 5 engorgement were retained [36].

### Mosquito processing and virus assay

On day 14 post-feeding, individual mosquitoes were chilled to immobilize, then dissected and homogenized (legs/wings and body separately) in tubes containing a steel BB and 500μl of media [DMEM supplemented with 20% (vol/vol) FBS, 100 U/mL of penicillin, 100 μg/mL of streptomycin, and 0.5 mg/mL amphotericin B (Sigma Aldrich, St. Louis, MO, USA)] and frozen at -80°C. Homogenate was assayed on C6/36 cells for the presence of SPONV antigen by an indirect fluorescent antibody (IFA) test using hyperimmune mouse ascitic fluid (HMAF) directed against the SPONV Chuku strain and a commercial fluorescein isothiocyanate-conjugated goat antimouse immunoglobulin G (Sigma Aldrich, St. Louis, MO, USA) [37,38].

### Statistical analyses

For each mosquito species and virus strain, the Wilson-Brown method [39] implemented in GraphPad Prism 7 (GraphPad Software, Inc, La Jolla, CA, USA) was used to calculate 95% confidence intervals for the percentage of mosquitoes with detectable infection and percentage of mosquitoes with disseminated infection.

## Results

### Genetic analyses

**Fig 1:**
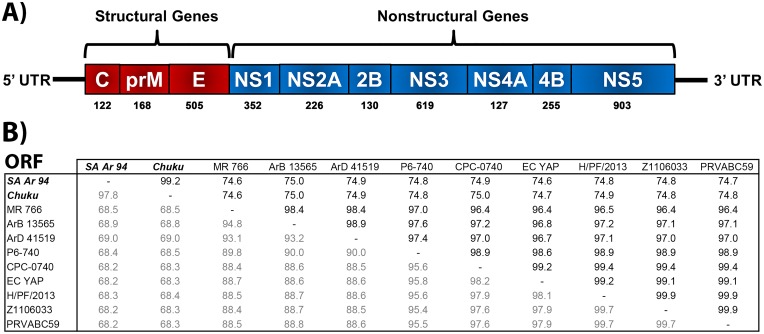
Genome structure and pairwise comparison of the open reading frame (ORF) of Spondweni (SPONV) and Zika (ZIKV) viruses.* A) SPONV genome organization: capsid (C), premembrane/membrane (prM), envelope (E), NS1, NS2A, NS2B, NS3, NS4A, NS4B and NS5. Numbers indicate animo acids in each protein. B) Pairwise comparison of the ORF of SPONV and ZIKV strains. SPONV SA Ar 94; SPONV Chuku; ZIKV MR 766; ZIKV ArB 13565; ZIKV ArD 41519; ZIKV P6-740; ZIKV CPC-0740; ZIKV EC Yap; ZIKV H/PF/2013; ZIKV Z1106033; ZIKV PRVABC59. *Boldface type (upper diagonal) = Percent amino acid identity; Lightface type (lower diagonal) = Percent nucleotide identity.

The ORF of SPONV Chuku and SA Ar 94 strains displayed >98% nucleotide and amino acid identity to each other, whereas they displayed ~68% and ~75% percent nucleotide and amino acid identity to ZIKV (Fig 1). Next we compared nucleotide and amino acid identity in the individual genes of SPONV and ZIKV. The lengths of individual genes were determined by utilizing putative cleavage sites of ZIKV genes. The individual SPONV gene sizes were similar to ZIKV genes: C prM, NS1, NS4A, and NS5 were identical, whereas the E (505 vs. 504 amino acid), NS2A (226 vs. 217 amino acid), NS2B (130 vs. 122 amino acid), NS3 (619 vs. 617 amino acid) and NS4B (255 vs. 251 amino acid) were larger than ZIKV. The individual structural gene comparison of SPONV and ZIKV showed nucleotide and amino acid identity ranging from 61% to 68% and 64% to 72%, respectively, with the E gene displaying greater sequence identity (68% nucleotide and 72% amino acid) (S1 Fig). The nonstructural gene comparison displayed nucleotide and amino acid identity ranging from 59% to 73% and 58% to 82%, respectively. The NS4B and NS3 genes displayed the greater identity, 70% to 72% nucleotide and 81 to 82% amino acid (S2 and S3 Figs). The NS2A gene was the most divergent gene with 59% to 60% nucleotide and 58% to 59% amino acid identity between SPONV and ZIKV (S3 Fig).

### Mosquito infection and dissemination

Exposure to the SPONV Chuku strain by artificial infectious blood meal (5.1 log_10_ PFU/mL) did not result in any detectable infection or dissemination in any of the three mosquito species (Table 2). Exposure to the SPONV SA Ar 94 strain by artificial infectious blood meal (5.3 log_10_ PFU/mL) resulted in detectable infection in 8.3% of *Ae*. *aegypti* (Galveston) and 12.5% *Ae*. *aegypti* (Thailand), while only *Ae*. *aegypti* (Galveston) developed detectable disseminated infection (8.3%). Poor feeding rates (stages 1 to 3 engorgement) and high mortality experienced in *Ae*. *aegypti* (Thailand) and *Ae*. *albopictus* (Venezuela) mosquitoes exposed to the SPONV Chuku strain resulted in low numbers of experimentally exposed mosquitoes.

## Discussion

Prior to this study, only one SPONV strain had been sequenced, but its geographic origin and passage history were not reported [40]. Our analyses demonstrated that both SPONV strains sequenced in this study (Chuku and SA Ar 94) are genetically similar, but exhibit a high degree of nucleotide and amino acid divergence when compared to ZIKV strains from West Africa, East Africa, Southeast Asia, the Pacific Islands and the New World (Fig 1). The similarity between the two SPONV strains isolated in different geographic regions approximately 2.5 years apart indicates the possibility of continuous enzootic transmission and maintenance between Nigeria and South Africa, although interpretation is limited due to the lack of spatial and temporally spaced sequences (i.e. multiple isolates). ZIKV strains within each lineage, African and Asian, also exhibited a low degree of nucleotide divergence when compared to one another, as seen in previous work [11]. With the exception of the MR 766 ZIKV strain, neither SPONV strain nor any of the other eight ZIKV strains used in this study exhibited a deletion in the potential N-linked glycosylation site as reported in some ZIKV strains that had prior passage histories in mouse brains [11,22,31,41].

The susceptibility and dissemination to moderate doses of both SPONV strains in all three species was low or non-existent (Table 2). The Chuku strain caused no detectable infection or dissemination in any of species, while the SA Ar 94 strain was only observed to cause disseminated infection in *Ae*. *albopictus* Galveston (8.3%). Work by Bearcroft also failed to show transmission of the Chuku strain by *Ae*. *aegypti* [4]. Unlike SPONV, *Ae*. *aegypti* and *Ae*. *albopictus* have been incriminated as primary urban vectors of ZIKV [25–28,42–48]. Early work demonstrated that *Ae*. *aegypti* was a competent vector of ZIKV following feeding on an artificial infectious blood meal containing the MR 766 prototype strain, with three mosquitoes transmitting ZIKV to a single rhesus monkey 72 days post-exposure [27]. Since that time, numerous studies have shown that various geographically distinct strains of *Ae*. *aegypti* or *Ae*. *albopictus* mosquitoes exposed to ZIKV strains from either the African and Asian lineages exhibit a wide range of susceptibility and/or vector competence in these two mosquito species [25,26,28,43–48]. Although there have been reports that *Cx*. *quinquefasciatus* could be a potential vector of ZIKV [49,50], multiple vector susceptibility and/or competence studies using laboratory or field strains of *Cx*. *quinquefasciatus* or *Cx*. *pipiens* indicate that many geographically distinct populations are refractory to virus transmission [46,47,51–54]. These results are similar to our findings in both SPONV strains, where infection and dissemination was not detected in *Cx*. *quinquefasciatus*.

While there is little information on the potential sylvatic amplification and maintenance hosts of SPONV [2], intensive field studies carried out in areas of high SPONV transmission were able to narrow down or exclude potential host species [7,18]. Virus isolations and antibodies were not detected in any rodent or bird collected in Ndumu, South Africa in 1958 [7]. These findings led the authors to speculate that it was unlikely these species were involved in the amplification and maintenance of the SPONV. Later experimental work supported this hypothesis, when six African rodent species in different genera failed to develop viremia following experimental inoculation with SPONV [55]. During the course of these early field studies antibodies were detected in domestic livestock [7,56], however the ability of these species to develop viremia remains unknown. Similar to ZIKV, experimental work demonstrated that SPONV can infect non-human primates [18,57]. While little is known in regards to the species of non-human primates SPONV may infect in nature, considerable information exists for ZIKV. In Africa, ZIKV has been isolated and/or a serological response to prior infection has been observed in numerous non-human primate species including members of the genera *Cercocebus*, *Cercopithecus*, *Colobus*, and *Erythrocebus* [11,13,17]. Based on historic reports and its close genetic relationship with ZIKV, SPONV may be maintained and transmitted in a sylvatic cycle involving non-human primates and mosquitoes.

Unlike ZIKV, which has a broad geographic distribution [11,13–15], SPONV isolations and seroprevalence have thus far been confined to Africa (Table 1). While it is possible that the differences in the geographic distribution of ZIKV and SPONV are a result of prior infection either virus resulting in a refractory status among amplification hosts, another explanation is that different sylvatic vector species are involved in the transmission of these two viruses. To date, the majority of SPONV isolations have been made in *Ae*. *circumluteolus* mosquitoes collected in Southern Africa [7,20,21], with experimental work demonstrating this species is capable of virus transmission up to 84 days following exposure to 7.1 log_10_ PFU/mL of virus [20,58]. Isolations made from other sylvatic mosquito species are considerably less common [7,18,20,21], which may be a result of sampling bias [20]. In contrast, the commonly incriminated sylvatic vectors of ZIKV in sub-Saharan Africa are *Ae*. *africanus*, *Ae*. *furcifer*, *Ae*. *opok*, *Ae*. *vittatus*, and *Ae*. *luteocephalus* [11,13]. Mosquito collections and subsequent virus isolation attempts over a number of years by laboratories in sub-Saharan Africa yielded isolations of SPONV from eight species of mosquitoes in the genera *Aedes*, *Culex*, *Eretmapodites*, and *Mansonia* (Table 1), while ZIKV has been isolated in 20 species in the genera *Aedes*, *Anopheles*, *Eretmapodites*, and *Mansonia* [11]. Although many of these species are found in the same regions where both SPONV and ZIKV have been isolated, both viruses have only been isolated in two species, *Ae*. *fowleri* and *Ma*. *uniformis*. Ultimately, further studies are needed to determine the potential for sylvatic mosquito species to transmit both ZIKV and SPONV.

Our study has some limitations. We only had access to two SPONV strains whose prior passage histories are obscure, as such, passage associated mutations could be present. Based on ZIKV non-human primate and human viremia data [59,60], we choose a virus dose that would provide approximately 100 virus particles per 0.1 ul of infectious blood to experimental mosquitoes–what we concluded would constitute a moderate virus dose. As such, a higher virus dose may result in infection and dissemination in these species. While the presence of infectious virus was demonstrated by plaque titrating infectious blood meals, (5.1 log_10_ PFU/mL Chuku strain and 5.3 log_10_ PFU/mL SA Ar 94 strain), some studies have shown a decrease in infection and subsequent transmission among flaviviruses using infectious blood meals that have utilized freeze-thawed virus [61,62]. Finally, it is important to note that caution should be exercised regarding the over interpretation of the results of vector susceptibility/competence studies, as variation in virus strains and/or vector competence between geographically distinct mosquito populations has been reported in other arboviruses [63,64].

Previous to the Ninth Report of the International Committee on the Taxonomy of Viruses (ICTV) [65], SPONV was considered a species of the genus *Flavivirus*, family *Flaviviridae*, and both SPONV and ZIKV were considered members of the Spondweni serogroup [2]. According to the current report, SPONV has now been categorized as a member of the genus *Flavivirus* that has not been approved as a species. SPONV clearly exhibits a greater nucleotide (~ 32%) and amino acid (~25%) divergence from ZIKV as has been previously reported (Fig 1) [31]. This is particularly evident when comparing individual proteins rather than the entire ORF (S1, S2 and S3 Figs). Comprehensive historic work using neutralization, hemagglutination-inhibition, complement fixation and antibody absorption tests also differentiate SPONV and ZIKV as distinct viruses based on limited cross-reactivity [2,3,17,66,67]. Furthermore, both viruses exhibit differences in vector associations, ecology, and geographic distribution. These data suggest that although both SPONV and ZIKV are related, they are separate species.

In conclusion, this study determined the genetic relationship between two SPONV strains, as well as their relationship to nine representative African and Asian lineage ZIKV strains. *Aedes aegypti*, *Ae*. *albopictus* and *Cx*. *quinquefasciatus* mosquitoes exhibited poor infection and virus dissemination rates following exposure to moderate oral infectious doses of both SPONV Chuku and SA Ar 94 strains, indicating a low potential for these species to serve as vectors. Based on these results, SPONV probably has limited potential for emergence into urban cycles that are characteristic of other flaviviruses such as Zika, yellow fever and dengue viruses. Nucleotide and amino acid divergence coupled with differences in geographic distribution, ecology and vector species support previous reports that SPONV and ZIKV are separate species.

## Supporting Information

S1 Table Spondweni virus sequencing primers. (DOCX)

S1 Fig Pairwise comparison of the structural proteins of Spondweni (SPONV) and Zika (ZIKV) viruses.* Capsid (C), premembrane/membrane (prM), and envelope (E). SPONV SA Ar 94; SPONV Chuku; ZIKV MR 766; ZIKV ArB 13565; ZIKV ArD 41519; ZIKV P6-740; ZIKV CPC-0740; ZIKV EC Yap; ZIKV H/PF/2013; ZIKV Z1106033; ZIKV PRVABC59. *Boldface type (upper diagonal) = Percent amino acid identity; Lightface type (lower diagonal) = Percent nucleotide identity.
(TIF)

S2 Fig Pairwise comparison of the non-structural proteins NS2b, NS3, and NS5 of Spondweni (SPONV) and Zika (ZIKV) viruses.* SPONV SA Ar 94; SPONV Chuku; ZIKV MR 766; ZIKV ArB 13565; ZIKV ArD 41519; ZIKV P6-740; ZIKV CPC-0740; ZIKV EC Yap; ZIKV H/PF/2013; ZIKV Z1106033; ZIKV PRVABC59. *Boldface type (upper diagonal) = Percent amino acid identity; Lightface type (lower diagonal) = Percent nucleotide identity.
(TIF)

S3 Fig Pairwise comparison of the non-structural proteins NS1, NS2a, NS4a, and NS4b of Spondweni (SPONV) and Zika (ZIKV) viruses.* SPONV SA Ar 94; SPONV Chuku; ZIKV MR 766; ZIKV ArB 13565; ZIKV ArD 41519; ZIKV P6-740; ZIKV CPC-0740; ZIKV EC Yap; ZIKV H/PF/2013; ZIKV Z1106033; ZIKV PRVABC59. *Boldface type (upper diagonal) = Percent amino acid identity; Lightface type (lower diagonal) = Percent nucleotide identity.
(TIF)
