# Supplementary material for: Genetic Characterization of Spondweni and Zika Viruses and Susceptibility of Geographically Distinct Strains of Aedes aegypti, Aedes albopictus and Culex quinquefasciatus (Diptera: Culicidae) to Spondweni Virus
Source: PLoS Negl Trop Dis. 2016 Oct 26;10(10):e0005083. doi: 10.1371/journal.pntd.0005083 (PMC5082648; doi:10.1371/journal.pntd.0005083)
Supplement: S1 Table — (DOCX) [file pntd.0005083.s001.docx]

Supplementary Table 1. Spondweni virus sequencing primers.

| _Region_ | _Sense Primer_ | _Antisense Primer_ |
| --- | --- | --- |
| _6-886_ | _AAACCCAAAAAGAGCCGGTAGC_ | _CAATTCCAATGCATCTGATGC_ |
| _6-1214_ | _AAACCCAAAAAGAGCCGGTAGC_ | _CAAGTGACAATGCTTCCTTTTCC_ |
| _687-1504_ | _ATCATCGACGTGGCTTGAATCC_ | _GCCAATGCTTGTTGTTCATGG_ |
| _1000-2214_ | _CTGGTGACAACGACCGCAAGT_ | _CACCTGGTGGACAAACTTTCC_ |
| _1333-2170_ | _ACCAATCACCAACACGACAAGG_ | _CAGAGCCAAAGTCCCAAGC_ |
| _1972-2799_ | _AGTGGAGCAAACTCAAAGATGATGG_ | _GGTGTCACCATCTACCACAAAGG_ |
| _2002-3312_ | _ATTGACCCTCCGTTTGGTGATTC_ | _TTCATTGATTACCCTTCCACTAGC_ |
| _2634-3458_ | _GCCATTGACAGTCGTCGTTGG_ | _TCCATGTGGTCTGTGCTTCC_ |
| _3102-4357_ | _GCTGATCATTCCACGTGGCTTAG_ | _TGTCGTCGAGAGCCACATCC_ |
| _3291-4097_ | _TAGTGGAAGGGTAATCAATGAATGG_ | _AACAAGAGGAGGCCAACCAA_ |
| _3903-4720_ | _CGCTATGCTGTCACCCATGC_ | _CGTTTCCCCAGTATGGATCTAGG_ |
| _4161-5376_ | _TGCAATTGTGGGCGGACTAAC_ | _TGTGAAGTGGGCCTCATCC_ |
| _4540-5417_ | _GAAGTGAAAAAAGGGGAAACAACG_ | _CTTGTTGCTATGTACCCTCTTGC_ |
| _5184-6538_ | _AGTGGTGGCAGCTGAGATGG_ | _TGAGGAGCAGGATGGTTTCC_ |
| _5247-6069_ | _AGTTTCAGCCACCCATGATGG_ | _TTCTATTGCCGAGACCTTTCC_ |
| _5873-6696_ | _CTCAACGCAGAGGAAGAATTGG_ | _TATGATCACCACACAAGCTATGC_ |
| _6338-7582_ | _TCAAGGAGTTTGCTGCAGGAAAG_ | _CGCCCCTTTTCTTCATGATACC_ |
| _6500-7341_ | _CACAGCTCCCTGAGACAATGG_ | _CTGGCCCATCTTCTTTTCG_ |
| _7152-7957_ | _GATCGTGGCTCTCGTGATGC_ | _TGAGTCGCACTATGTTCCAACC_ |
| _7281-8498_ | _TGTGGTAACTGACATAGACCCAATC_ | _TGGTAATGCCATGTCCTGTAGG_ |
| _7757-8590_ | _CAAAGCTGCGATGGATGG_ | _AGCTCAATGCATCCCATGG_ |
| _8290-9514_ | _CAGCTGCTCATGCACAGAATG_ | _TGACTTTCCATGGGTTTTGG_ |
| _8420-9237_ | _GGATTGAGCGGTTGAGAAAGG_ | _GCGCGCCTCATTTTCTAGG_ |
| _9054-9856_ | _CCTCAATGAAGACCACTGGTTAAGC_ | _AAGACTTGCCCAAGCATGC_ |
| _9319-10164_ | _GTCTTGAGACCAGCACCACAAGG_ | _TGCCCATGTGCTTCTTGG_ |
| _9567-10219_ | _TGGAGATGACTGTGTCGTGAAACC_ | _CTTCAGTTTCGCCGATTGC_ |
