# Supplementary figures and images for: Genetic Characterization of Spondweni and Zika Viruses and Susceptibility of Geographically Distinct Strains of Aedes aegypti, Aedes albopictus and Culex quinquefasciatus (Diptera: Culicidae) to Spondweni Virus
Source: PLoS Negl Trop Dis. 2016 Oct 26;10(10):e0005083. doi: 10.1371/journal.pntd.0005083 (PMC5082648; doi:10.1371/journal.pntd.0005083)

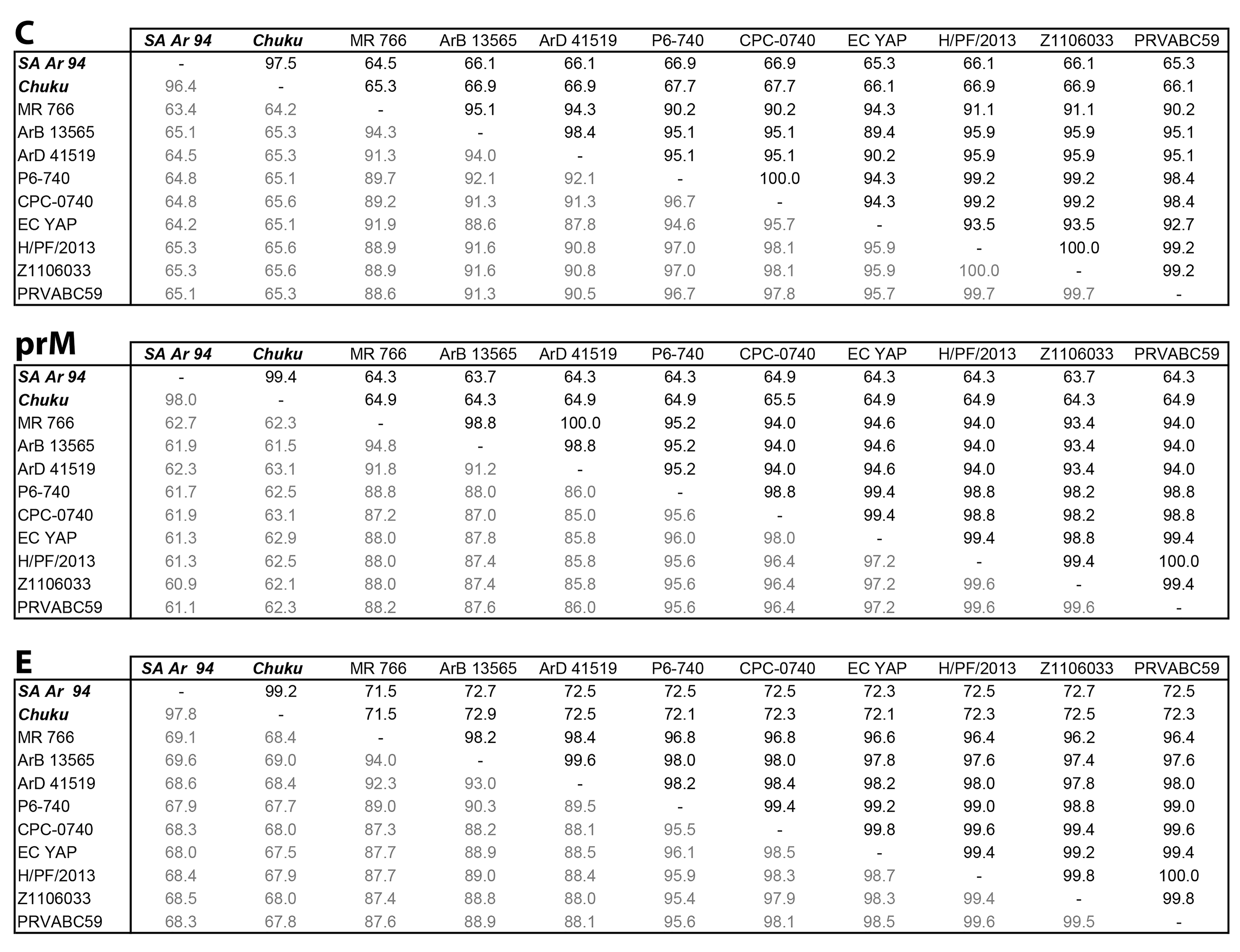

Supplement: S1 Fig — Capsid (C), premembrane/membrane (prM), and envelope (E). SPONV SA Ar 94; SPONV Chuku; ZIKV MR 766; ZIKV ArB 13565; ZIKV ArD 41519; ZIKV P6-740; ZIKV CPC-0740; ZIKV EC Yap; ZIKV H/PF/2013; ZIKV Z1106033; ZIKV PRVABC59. *Boldface type (upper diagonal) = Percent amino acid identity; Lightface type (lower diagonal) = Percent nucleotide identity. (TIF) [file pntd.0005083.s002.tif]

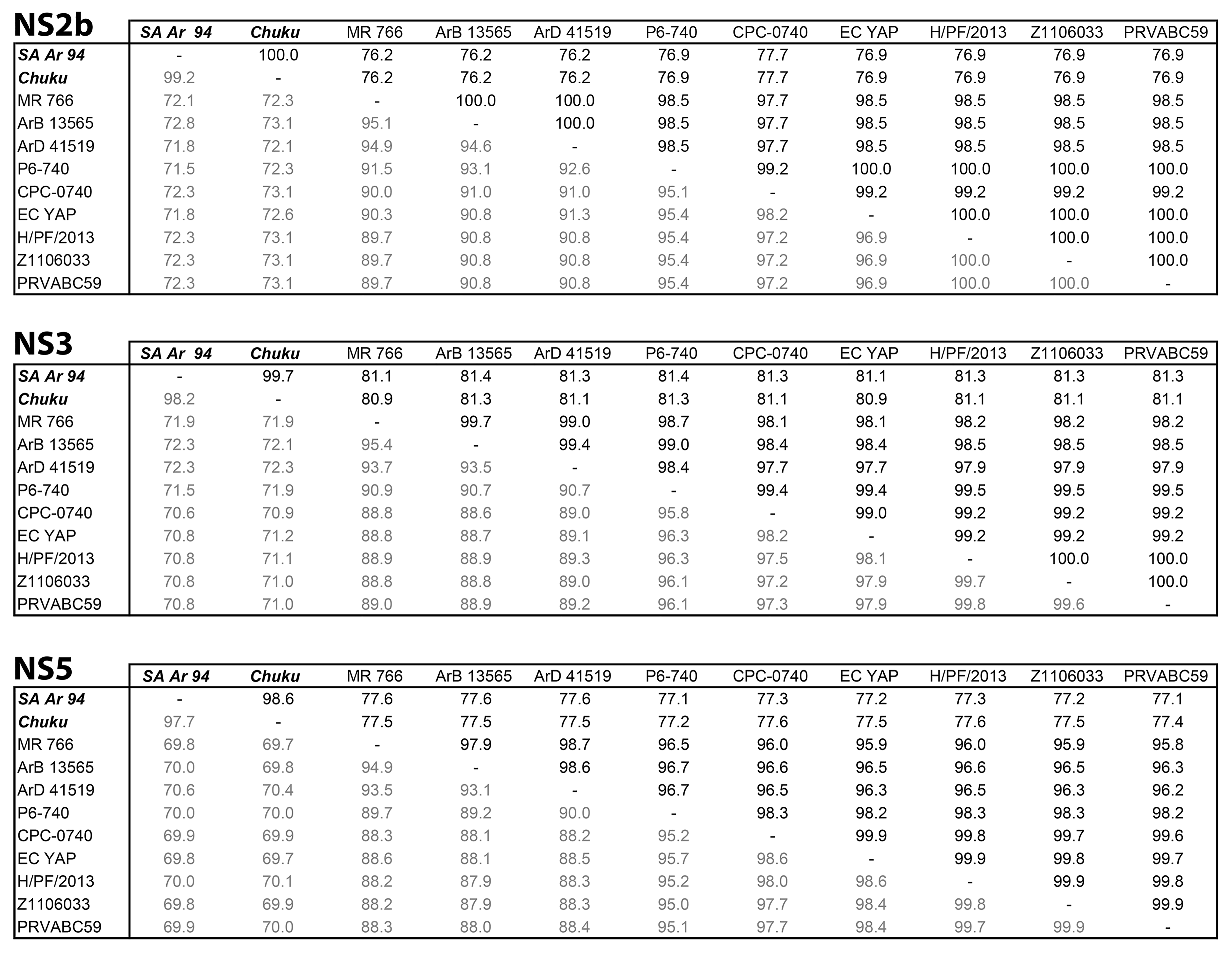

Supplement: S2 Fig — SPONV SA Ar 94; SPONV Chuku; ZIKV MR 766; ZIKV ArB 13565; ZIKV ArD 41519; ZIKV P6-740; ZIKV CPC-0740; ZIKV EC Yap; ZIKV H/PF/2013; ZIKV Z1106033; ZIKV PRVABC59. *Boldface type (upper diagonal) = Percent amino acid identity; Lightface type (lower diagonal) = Percent nucleotide identity. (TIF) [file pntd.0005083.s003.tif]

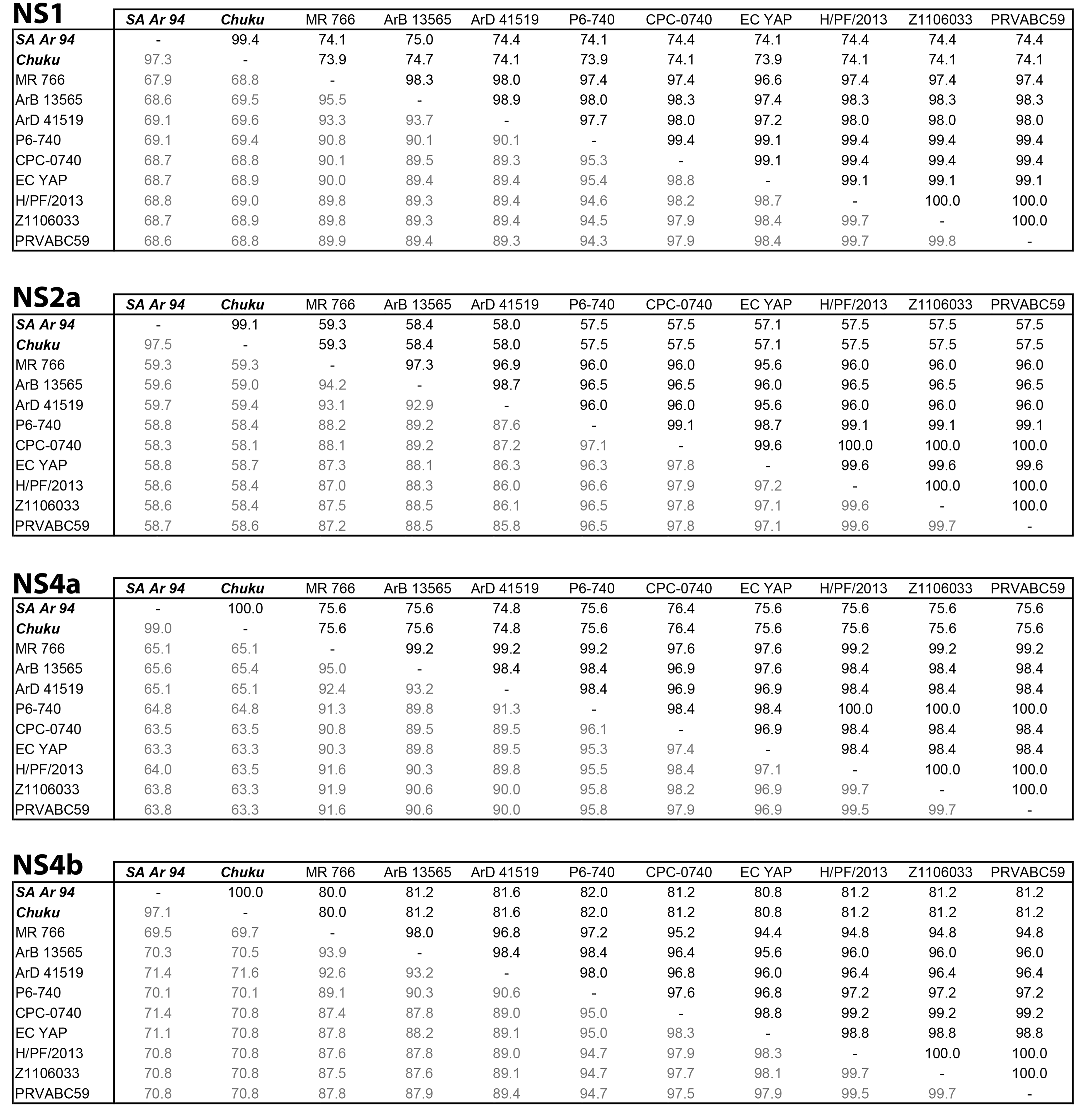

Supplement: S3 Fig — SPONV SA Ar 94; SPONV Chuku; ZIKV MR 766; ZIKV ArB 13565; ZIKV ArD 41519; ZIKV P6-740; ZIKV CPC-0740; ZIKV EC Yap; ZIKV H/PF/2013; ZIKV Z1106033; ZIKV PRVABC59. *Boldface type (upper diagonal) = Percent amino acid identity; Lightface type (lower diagonal) = Percent nucleotide identity. (TIF) [file pntd.0005083.s004.tif]
